# siRNA and shRNA screens advance key understanding of host factors required for HIV-1 replication

**DOI:** 10.1186/1742-4690-6-78

**Published:** 2009-08-27

**Authors:** Kin-Hang Kok, Ting Lei, Dong-Yan Jin

**Affiliations:** 1 Department of Biochemistry, the University of Hong Kong, 21 Sassoon Road, Pokfulam, Hong Kong, PR China

## Abstract

A recent RNAi screen used a genome-wide shRNA library to search for cellular factors required for HIV-1 replication. This work complements three other siRNA-based screening studies and potentially opens the door to the discovery of factors that are important for HIV-1 replication in physiological host cells such as T lymphocytes. shRNA screens can be further improved, and they could promise to unravel new pathways and new facets of virus-cell interactions.

## Commentary

**Figure 1:**
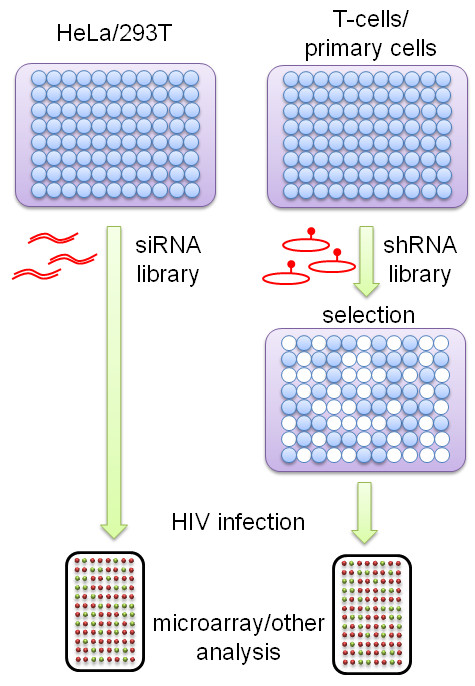
**A comparison between siRNA- and shRNA-based screens for HIV-1 replication cofactors**. See text for additional details.

The advent of RNAi-based whole-genome screens in mammalian cells provides a new impetus to the search of host cell factors needed for HIV replication [1,2]. Three screens that used siRNA pools to identify cellular proteins important in HIV-1 replication were reported in 2008, and a meta-analysis of these studies has been published recently [3-6]. One shortcoming to these reported screens is the use of HeLa or HEK293T cells that are not physiological substrates for infection by HIV-1. In addition, the use of a pseudotyped virus or a mutated strain of HIV-1 also limits the interpretability of some of the results. With this backdrop, a recently published genome-wide shRNA-screening performed in Jurkat T lymphocytes for cellular genes that contribute to HIV-1 replication (Figure 1) advances the field by extending the functional screening for cellular factors from attached epithelial/fibroblast cells to suspension T-cells [7].

In the shRNA loss-of-function screen, Jurkat cells are transduced with a lentiviral vector-based shRNA library. The lentivector is derived from feline immunodeficiency virus and is pseudotyped with the vesicular stomatitis virus G protein. A major advantage that makes this scheme attractive is its potential application to cells that can be physiologically infected by HIV-1, including primary T cells and macrophages. In addition, it is noteworthy that the transduced Jurkat cells have been selected for shRNA-expression for extended duration before being subject to HIV-1 infection. This pre-infection selection for shRNA-expression serves to eliminate those shRNA-cell clones which are silenced for a gene whose knock down dramatically affects cell growth or survival. The pre-selection procedure thus significantly reduces the number of false positive genes identified in the screening. Unlike siRNAs, the activity of shRNAs in the cell is not transient, but is long-lasting. Because shRNAs are stably expressed, infection of cell clones with HIV-1 can be initiated at any desired time. Thus, HIV-1 infection can be performed long after the shRNA has silenced transcripts coding for long-lived proteins; these long half-life proteins generally cannot be depleted efficiently by transiently-transfected siRNAs. For the above reasons, shRNA screens have certain inherent and nuanced advantages over siRNA screens in selected settings.

Although four screens have been performed to date [3-7], the search for HIV-1 replication cofactors appears to be far from saturated. Overlaps among the genes identified in the four screens are scarce [7]; and some well known HIV-1 cofactors such as the Sp1 transcription factor, which drives LTR-dependent expression of viral genes, were not identified [6]. This lends credence to the notion that the approaches were not exhaustive. The goal of siRNA and shRNA screens is to identify all possible HIV-1 cofactors. For productive replication to occur, HIV-1 has to switch on and off many cellular pathways. Plausibly, at different stages of infection HIV-1 would hijack different cofactors to modulate the same cellular function for its own benefit. Thus, even an essential cofactor could become non-essential in a different experimental context (e. g., at an exceedingly high multiplicity of infection). That is to say, the quantitative differences in critical assay parameters such as multiplicity and duration of infection might actually affect the qualitative outcomes of a siRNA or shRNA screen. In addition, off-target effects, differences in cell types and differences in how the primary data sets are filtered have also been suggested to account for the identification of the many different HIV-1 cofactors in the screens [1].

In the shRNA screen of Yeung et al., although the removal of cell clones that did not survive puromycin selection helped to reduce the number of false positive genes whose loss would globally inhibit cell growth or survival, rather than specifically affect HIV-1 replication, this step which eliminated more than 80% of the transduced cell clones might falsely miss those HIV-1 cofactors that are also simultaneously important for cell growth and/or survival. This issue has to be addressed in future screens. Thus, as the search continues, many more HIV-1 cofactors from different cell types are likely to emerge from further targeted si-/sh-RNA screens. Collectively, the four initial RNAi screens have already implicated new pathways that were not known previously to play critical roles in HIV-1 replication [8]. For example, HIV-1 cofactors have been identified as components of the mediator complex that regulates transcription [9], the nuclear pore complex that regulates macromolecular entry and egress, and the Golgi apparatus that specifies protein processing. While the mediator complex is thought to support Tat-dependent transcriptional activation of HIV-1 long terminal repeats (LTR), the nucleoporins and Golgi proteins could be critically involved in the intracellular transport and processing of viral nucleic acids and proteins (Table 1). Given the wide ranging functions of host cofactors identified in the four screens, the jury is still out as to which newly identified factors are of preeminence in the HIV-1 life cycle. As mentioned above, it would not be surprising that depending on the cell type and the experimental context, different cellular factors may be implicated as being rate-determining for HIV-1 replication. For example, the identification of different nucleoporins in different screens suggests that HIV-1 might employ different components of the nuclear pore complex to facilitate nuclear import/export of viral nucleic acids and proteins under different circumstances.

**Table 1:** Selected HIV-1 cofactors identified in the siRNA and shRNA screens

**Gene name**	**Protein function**	**Possible role in HIV-1 life cycle**
MED4	mediator complex subunit	Tat-dependent activation of LTR
MED7	mediator complex subunit	Tat-dependent activation of LTR
MED14	mediator complex subunit	Tat-dependent activation of LTR
MED28	mediator complex subunit	Tat-dependent activation of LTR
NUP98	nucleoporin	nuclear import/export of viral nucleic acids and proteins
NUP155	nucleoporin	nuclear import/export of viral nucleic acids and proteins
NUP210	nucleoporin	nuclear import/export of viral nucleic acids and proteins
GCC1	peripheral Golgi protein	processing of viral proteins
GOLM1	Golgi transmembrane protein	processing of viral proteins
GOSR2	Golgi membrane protein	processing of viral proteins

HIV-1 infection has been suggested to exert a suppressive effect on miRNA processing and RNA-silencing [10-16]. Indeed, the forced overexpression of shRNA and siRNA in cells might also have the potential to exhaust the cellular machinery for RNA silencing [17], complicating the interpretation of biological outcomes. Whether these influences might significantly affect the siRNA and shRNA screens for HIV-1 cofactors remains to be determined. We note, however, that the expression of a single shRNA from a lentiviral vector is unlikely to overwhelm the cell's RNAi machinery. Moreover, when shRNA-cell clones are infected by HIV-1, the shRNA-silencing of targeted mRNAs would have already been completed and would be unlikely influenced by the effects of infection on RNAi activities.

One important direction for improving the shRNA approach is to use inducible expression systems to express the shRNAs. In this regard, a deoxycycline-inducible retroviral vector has already been used successfully to construct a shRNA expression library [18]. Inducible expression of shRNAs might also help overcome potential adaptation by the cell to shRNAs, and the premature elimination of many shRNA-expressing cell clones. A further improvement to control for the possibility that some HIV-1 cofactors might also be required for the full expression of the feline immunodeficiency virus derived lentiviral vector would be to use alternate expression formats such as adenoviral vectors.

The technology of shRNA-based screening is still in its infancy [19,20]. These early reports of siRNA/shRNA screens for HIV-1 replication cofactors are therefore not the beginning of the end, but the end of the beginning of a new era in the search for host factors required for HIV-1 replication. Future screens will improve the stringency of the selection, expand upon the cell types being analyzed, and devise better strategies to address false positive/negative candidates. Furthermore, more specific questions in HIV-1 life cycle might also be addressed with siRNA and shRNA screens. For example, screens can be used to identify all the cofactors required for a specific process in HIV-1 replication such as fusion, viral entry, Tat-dependent transcription or integration. Synthetic lethal screens may also be employed to shed light on the functional interaction between different cofactors. In the years to come, the four recently reported complementary siRNA and shRNA approaches will likely be regarded as setting an important milestone for our understanding of host cell – HIV-1 interaction.
